# Assessment of dehydroepiandrosterone sulphate (DHEAS) and cortisol levels in saliva and their correlation to cervical vertebrae maturation method in males and females at different growth stages: a clinical study

**DOI:** 10.1590/2177-6709.28.4.e2322277.oar

**Published:** 2023-08-25

**Authors:** Nikita Dattatraya GAVATE, Smitha SHETTY, Rabindra S. NAYAK, Vinay K., Anjali NARAYAN, Chaitra K. R.

**Affiliations:** 1 Mathrusri Ramabai Ambedkar Dental College & Hospital, Department of Orthodontics (Bangalore/India).

**Keywords:** Cervical Vertebrae Maturation Method, Puberty, Biomarkers

## Abstract

**Objective:**

The aim of this *in-vivo* study was to assess the salivary dehydroepiandrosterone sulphate (DHEAS) and cortisol levels, and their correlation to the Cervical Vertebrae Maturation method (CVM) in males and females at pre-pubertal, pubertal and post-pubertal growth stages.

**Methods:**

48 patients (24 males, 24 females) who were to undergo routine orthodontic treatment were screened according to the inclusion and exclusion criteria. Then subjects were grouped according to CVM stages, using lateral cephalogram, in pre-pubertal, pubertal and post-pubertal groups. Unstimulated saliva from the selected subjects was collected. DHEAS and cortisol levels in the salivary samples were estimated by Enzyme-Linked Immunosorbent assay (ELISA). Then they were compared to Cervical Vertebrae Maturation Method stages. One-way ANOVA test followed by Tukey’s *post-hoc* test was used to compare the salivary DHEAS and cortisol levels between different CVM stages in males and females. Independent Student *t*-test was used to compare the mean salivary DHEAS and cortisol levels between different males and females in each CVM stage.

**Result:**

There was a progressive increase in salivary DHEAS and cortisol concentration as skeletal maturation progressed from CVM stages 1 and 2, CVM stages 3 and 4, reaching the highest value at CVM stages 5 and 6. Their levels were higher in males than females.

**Conclusion:**

The salivary DHEAS and cortisol levels can be useful as a potential indicator of skeletal maturation, to aid in the assessment of pubertal status.

## RESUMO

**Objetivo:**

O objetivo deste estudo *in vivo* foi avaliar os níveis salivares de sulfato de dehidroepiandrosterona (DHEAS) e de cortisol, e sua correlação com o método de maturação das vértebras cervicais (CVM) em homens e mulheres nas fases de crescimento pré-puberal, puberal e pós-puberal.

**Métodos:**

48 pacientes (24 homens, 24 mulheres) que se submeteriam a tratamento ortodôntico de rotina foram selecionados de acordo com os critérios de inclusão e exclusão. Em seguida, usando telerradiografia lateral, os indivíduos foram agrupados de acordo com os estágios CVM, em grupos pré-puberal, puberal e pós-puberal. Foi feita coleta de saliva não estimulada nos indivíduos selecionados. Os níveis de DHEAS e cortisol nas amostras salivares foram avaliados pelo teste ELISA (*Enzyme-Linked Immunosorbent assay*). Em seguida, foram comparados aos estágios do método CVM. O teste ANOVA de uma via seguido pelo teste *post-hoc* de Tukey foi usado para comparar o DHEAS salivar e os níveis de cortisol entre os diferentes estágios de CVM em homens e mulheres. O teste *t* de Student independente foi usado para comparar a média de DHEAS salivar e os níveis de cortisol entre diferentes homens e mulheres em cada estágio de CVM.

**Resultados:**

Houve um aumento progressivo no DHEAS salivar e na concentração de cortisol à medida que a maturação esquelética progrediu dos estágios CVM 1 e 2, para os estágios CVM 3 e 4, atingindo o valor mais alto nos estágios CVM 5 e 6. Seus níveis foram maiores nos homens do que nas mulheres.

**Conclusões:**

Os níveis salivares de DHEAS e cortisol podem ser úteis como um potencial indicador de maturação esquelética, para auxiliar na avaliação do estado puberal.

## INTRODUCTION

Appropriate timing of prevention, interception or correction of skeletal malocclusion is key to success in Orthodontics. This assessment of timing is based on the knowledge of growth and development of the craniofacial complex. The rationale of orthodontic treatment is based on modification of growth during the greatest craniofacial growth period or the option of camouflage and orthognathic surgery at a later period.^1^

The assessment of skeletal growth and maturation involves the use of several skeletal and dental indicators including: increase in body height,^2^ dental development and eruption,^3^ ^,^ ^4^ cervical vertebral maturation (CVM),^5^ ^,^ ^6^ ^,^ ^7^ skeletal maturation of the hand and wrist,^8^ ^,^ ^9^ frontal sinus development.^10^ Among them, CVM method assessed using lateral cephalogram is frequently used, as it reduces the additional radiation exposure attributable to a hand-wrist radiograph. Certain biomarkers such as alkaline phosphatase,^11^ insulin-like growth factor (IGF),^12^ serum parathyroid hormone-related protein (PTHrP),^13^ dehydroepiandrosterone sulphate (DHEAS)^14^ and cortisol have also been attempted to be used as maturity indicators. 

There are several ways to assess their levels, such as blood, plasma, gingival cervical fluid (GCF), saliva, etc. But salivary sampling has the advantages of ease of collection and non-invasiveness in individuals, and can be done chair-side also.^15^ Also medically compromised patients who should avoid X-ray radiation will benefit from salivary sample collection.

Puberty is primarily a neuroendocrinal event, with the pituitary and hypothalamus playing major roles in its initiation, which together are called as gonadostat.^16^ DHEA and Cortisol released from adrenal cortex have important implication on pubertal growth through their influence on Hypothalamus-Pituitary-Adrenal axis (HPA axis).

DHEA is released by adrenal glands into the circulation as the sulfated form - DHEAS which constitutes of 99% of circulating DHEA which are precursors for androgens- testosterone and dihydrotestosterone.^16^ The onset of production of DHEAS from the reticularis zona of the adrenal cortex starts at around the age of six, increasing gradually reaching the peak at around 20-30 years, and declines to 20-30% of the peak levels by around 70-80 years. Unlike DHEA, DHEAS does not show any diurnal variation.^14^

Cortisol is the principle of glucocorticoid, which is produced and secreted by the zona fasciculata of the adrenal cortex. It circulates in the plasma mostly in bound form to globulin or albumin, showing sharp rise at pubertal spurt and gradual postpubertal increase with aging. Trace amounts of cortisol are found in saliva due to its properties of low molecular weight and lipophilic nature. Studies have reported high correlations between serum and salivary cortisol levels.^17^ Cortisol follows a circadian rhythm, reaches a peak in early morning and lowest levels at night.^18^

As DHEAS and cortisol play a major role in the initiation of puberty, it would be more appropriate to measure the level of these biomarkers to assess the maturational stage of pubertal growth spurt. Hence, this study aims to assess the salivary dehydroepiandrosterone sulphate (DHEAS) and cortisol levels, and their correlation to the CVM method in males and females at pre-pubertal, pubertal and post-pubertal growth stages.

## MATERIAL AND METHODS

a. Material used for the study: Mouth mirror, Explorer, Tweezer, X-ray machine (KODAK 8000C Carestream), X-ray film (KODAK) (8×10-in), X-ray Viewer, Tracing sheets (8×10-in), Tracing pencil (0.3 mm), 5 ml Locked Petri Dishes, Buffer solution, -80°C Lab Freezer, DHEAS and Cortisol ELISA kit.

b. Sample size estimation: The sample size has been estimated using the GPower v. 3.1.9.2 software.

Considering the effect size to be measured (f) at 48%, power of the study at 80% and the margin of the error at 5%, the total sample size needed was 45. Each group should consist of 15 samples. 

Hence according to sample size estimation, 16 patients(8 males and 8 females) were selected in each group for evaluation. Patients were grouped based on lateral cephalogram evaluation, as is routinely required for evaluation before undergoing orthodontic treatment. 

c. Sample selection: 48 subjects (24 males, 24 females) were selected from the patients undergoing treatment in the Department of Orthodontics and Dentofacial Orthopaedics, Mathrusri Ramabai Ambedkar Dental College & Hospital. This study was approved by the institutional ethics committee of the aforementioned institution. Patients with good to fair oral hygiene, with a Simplified Oral Hygiene Index^19^ score of 0 to 1.5 and good general health, were selected. Subjects with any systemic, endocrine disorders or under any medications were excluded from the study. 

Lateral cephalograms of the subjects undergoing orthodontic treatment, were taken with the cephalostat in the KODAK 8000C (Carestream, New York, USA) X-ray machine, at 80kV, 12 mA and an exposure time of 1 seconds using X-ray film (8×10”). The X-rays were traced and examined with help of X-ray viewer. X-rays with inadequate visualization of cervical vertebrae region were excluded, e. g. obscured cephalograms due to errors in the procedure or due to image distortion. 

The CVM method developed by McNamara, Bacetti and Franchi^7^ consists of visual analysis of the morphology of the bodies of the second (C2 - the odontoid process), third (C3) and fourth (C4) cervical vertebrae. The two factors studied in this method include: 1) Presence or absence of a concavity at the lower border of the body of C2, C3, and C4; and 2) Shape of the bodies of C3 and C4. 

Based on these parameters, the CVM stages are distinguished as six stages (CS1 to CS6), and patients within stages CS1 to CS6 were selected for the study. Stages CS1, CS2, stages CS3, CS4 and stages CS5, CS6 are considered as pre-pubertal, pubertal and post-pubertal stages, respectively. The subjects belonging to each of the six stages were segregated and then clustered into three groups of 16 subjects each, with each group representing one of the three growth phases, i.e. pre-pubertal, pubertal and post-pubertal (Table 1).

**Table 1:** Grouping according to the growth phase (pre-pubertal, pubertal and post-pubertal) and CVM stage (CS1 to CS6).

GROUPS	PUBERTAL STAGE	CVM STAGES	No. OF SUBJECTS
1	Pre-pubertal group	CS1, CS2	16 (8 males,8 females)
2	Pubertal group	CS3, CS4	16 (8 males,8 females)
3	Post-pubertal group	CS5, CS6	16 (8 males,8 females)

d. Salivary sample collection and assay: Unstimulated saliva from the selected subjects was collected using labeled petri dishes at a standard time of 9 AM, in order to exclude any diurnal variation in the salivary DHEAS and cortisol levels. The subjects were instructed not to use any intraoral creams or lotions and steroid inhalers. Also subjects were instructed to avoid alcohol consumption before 24 hours of collection, and to avoid eating or drinking anything or brushing or flossing the teeth 60 minutes before collection. 

Subjects were instructed to drool passively into the petri dishes by tilting the head forward. Collected saliva samples were mixed with buffer solution and the sample was freezed at -80°C within fours hours after collection. And then they were transferred to the laboratory in thermosealed box with ice packs for the assay. DHEAS and cortisol levels in the salivary sample were estimated by enzyme-linked immunosorbent assay (ELISA) and were compared to CVM method stages. The ELISA tests were conducted by Chromgene Biotech Lab Pvt. Ltd. (ISO registration no. 9001:2015), Bengaluru.

e. Statistical analysis: Statistical Package for Social Sciences (SPSS) for Windows v. 22.0 released 2013 (IBM Corp, Armonk, NY) was used to perform statistical analyses. Descriptive analysis includes expression of salivary DHEAS and cortisol levels in terms of mean and standard deviation for each CVM stage. One-way ANOVA test followed by Tukey’s *post-hoc* test was used to compare the salivary DHEAS and cortisol levels between different CVM stages in males and females. Independent Student *t*-test was used to compare the mean salivary DHEAS and cortisol levels between males and females in each CVM stage. The level of significance was set at *p*<0.05.

## RESULTS

**Figure 1:**
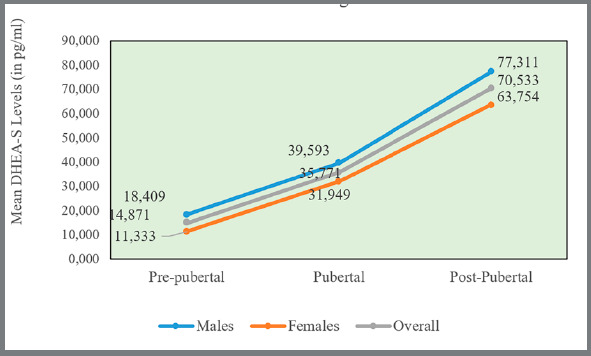
Mean salivary DHEAS levels ( in pg/ml ) between different CVM stages.

**Figure 2:**
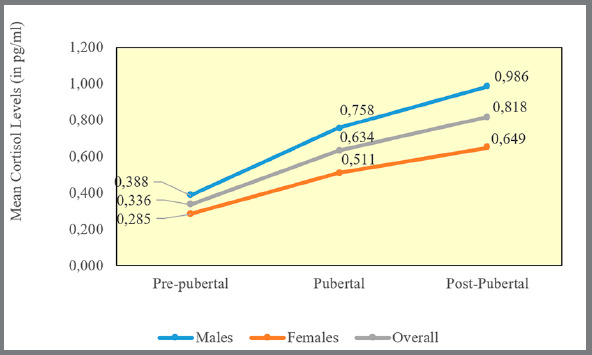
Mean salivary cortisol levels ( in pg/ml ) between different CVM stages.

The mean DHEAS values in each group were 14.871 (pre-pubertal), 35.771 (pubertal), and 70.533 pg/ml (post-pubertal) (Fig 1). And mean cortisol values in each group were 0.336 (pre-pubertal), 0.634 (pubertal), and 0.818 pg/ml (post-pubertal) (Fig 2). One-way ANOVA test suggests that the difference in the mean Salivary DHEAS levels (in pg/ml) (Table 2) and cortisol (Table 3) among males and females between different CVM stages was statistically significant (*p*<0.001). 

**Table 2:** One-way ANOVA test for comparison of mean salivary DHEAS levels (in pg/ml) in males and females.

Mean salivary DHEAS levels (in pg/ml)						
** ** ** **		Males		Females		***p*-value**
CVM stages	n	Mean	SD	Mean	SD	
Pre-pubertal	8	18.409	3.385	11.333	2.297	<0.001*
Pubertal	8	39.593	7.565	31.949	4.397	
Post-Pubertal	8	77.311	5.559	63.754	5.782	

* Statistically significant.

**Table 3:** One-way ANOVA test for comparison of mean salivary cortisol levels (in pg/ml) in males and females.

Mean salivary cortisol levels (in pg/ml)						
		Males		Females		
CVM stages	n	Mean	SD	Mean	SD	p-value
Pre-pubertal	8	0.388	0.061	0.285	0.035	<0.001*
Pubertal	8	0.758	0.089	0.511	0.042	
Post-Pubertal	8	0.986	0.137	0.649	0.034	

* Statistically significant.

Multiple pairwise comparison with Tukey’s *post-hoc* test (Tables 4 and 5) between different groups revealed that, the post-pubertal stage showed significantly highest mean salivary DHEAS and cortisol levels, as compared to pubertal and pre-pubertal stages (*p*<0.001). This infers that among males, mean salivary DHEAS and cortisol levels significantly increases with pubertal stage.

**Table 4:** Multiple comparison of mean difference in salivary DHEAS levels among males and females

(a) Groups	(b) Sub-groups	Mean difference (a-b) Males	Mean difference (a-b) Females	p-value
Pre-pubertal	Pubertal	-21.184	-20.616	< 0.001*
	Post-pubertal	-58.903	-52.421	< 0.001*
Pubertal	Post-pubertal	-37.719	-31.805	< 0.001*

* Statistically significant. Tukey’s *post-hoc* test.

**Table 5:** Multiple comparison of mean difference in salivary cortisol levels among males and females.

(a) Groups	(b) Sub-groups	Mean difference (a-b) Males	Mean difference (a-b) Females	p-Value
Pre-pubertal	Pubertal	-0.370	-0.226	< 0.001*
	Post-pubertal	-0.599	-0.364	< 0.001*
Pubertal	Post-pubertal	-0.229	-0.138	0.001*

* Statistically significant. Tukey’s *post-hoc* test.

Independent Student *t*-test (Table 6) indicated that the difference in the mean salivary DHEAS and cortisol levels (in pg/ml) in pre-pubertal and post-pubertal stages between males and females was statistically significant.

**Table 6:** Independent Student *t*-test.

Gender wise comparison			Mean salivary DHEAS levels (in pg/ml)				Mean salivary cortisol levels (in pg/ml)			
Groups	Sex	n	Mean	SD	Mean diff.	p-values	Mean	SD	Mean diff.	p-values
Pre-pubertal	Males	8	18.409	3.385	7.076	<0.001*	0.388	0.061	0.103	0.001*
	Females	8	11.333	2.297			0.285	0.035		
Pubertal	Males	8	39.593	7.565	7.644	0.03*	0.758	0.089	0.246	<0.001*
	Females	8	31.949	4.397			0.511	0.042		
Post-pubertal	Males	8	77.311	5.559	13.558	<0.001*	0.986	0.137	0.338	<0.001*
	Females	8	63.754	5.782			0.649	0.034		

* Statistically significant.

## DISCUSSION

Growth modification and modulation of the craniofacial skeletal pattern with orthopedic and functional appliances may be best accomplished when the growth rate is maximum during the pubertal growth spurt period. Earlier methods of assessing growth included physical stature, peak height velocity, and growth charts, which were in relation to chronological age.^14^ But it was observed that concordance of chronological age and maturational stage is not reliable. Hence, later skeletal maturation was used as an indicator of physical development and maturation, which led to the development of radiographic method of determining skeletal maturation. 

As lateral cephalogram is routinely used for orthodontic diagnosis, CVM method has become a frequently used skeletal maturity indicator, due to reduced additional radiation exposure attributable to a hand-wrist radiograph. Identification of changes in the size and shape of cervical vertebra and maturational standards were first developed by Lamparski^5^ in 1972 and further modified as an index, by Hassel and Farman^6^ in 1995, and as Cervical Vertebral Maturation (CVM) method by McNamara, Bacetti and Franchi^7^ in 2005. The disadvantages of this method includes difficulties in visualization of the subtle changes in the vertebrae, and due to incorrect neck posture while taking the radiograph, and blocking of cervical vertebrae due to the use of a thyroid collar.^20^

However, newer ways are being explored in the form of biochemical markers, with the use of various media such as blood, plasma, saliva and gingival cervical fluid (GCF), among others, which can act as an aid, as well as will avoid hazards of radiation exposure to growing patients.^15^ Since there are very few studies done to assess the salivary levels of biochemical markers in relation to skeletal maturity, the present study was done to find the feasibility of using the salivary DHEAS and cortisol as a reliable skeletal maturity indicator by finding a reliable correlation with CVM method. 

In present study, it was found that there is a progressive increase in salivary DHEAS and cortisol concentration as skeletal maturation progressed from CVM stages 1 and 2, CVM stages 3 and 4, reaching the highest value at CVM stages 5 and 6 among males and females (Tables 2 and 3, Figs 1 and 2).

Similar results were found in the study done by Srinivasan et al.^14^, in which serum DHEAS was correlated to hand-wrist stages. Their results also showed that low levels of DHEAS were present in the pre-pubertal group, with increased values in the pubertal group and the highest levels in the adult group, showing a gradual increase as maturation progresses. Several studies^21^ ^,^ ^22^ comparing the salivary DHEA levels of subjects and their pubertal status based on Tanner staging have noted higher mean levels in mid-post pubertal boys and girls than in pre-early pubertal boy and girls.

According to research, serum levels of DHEAS are high in the neonate, after which there is a decrease, then a rapid increase in the serum level from 7 years of age, with a gradual increase reaching the peak at around 20 to 30 years, and declines to 20-30% of the peak levels by around 70-80 years.^14^ Circulating concentrations of DHEAS are approximately 250 and 500 times higher than those of DHEA in women and men, respectively. Differences in concentrations of the two hormones are partly explained by metabolic clearance rate. DHEA is rapidly cleared from the blood at a rate of approximately 2000 L/day, while DHEAS has slower clearance of about 13 L/day. DHEA has a shorter half- life of 1 to 3 hours, while the half-life of DHEAS is 10 to 20 hours. The protein binding characteristics of the two hormones also differ and influence clearance rates; DHEA is weakly bound to albumin, whereas DHEAS is relatively strongly bound to albumin.^23^

Studies^24^ ^,^ ^25^ ^,^ ^26^ conducted to assess the relationship among cortisol, sex, and pubertal status showed that cortisol levels were age-dependent. After the age of six, cortisol levels correlated significantly with pubertal stages. No sex difference was found. In addition, cortisol morning levels and daily cortisol levels increased with body weight and body mass index. No circadian variation was evident before the age of 9 months and, after 1 year of age, salivary cortisol levels varied in a circadian way.^25^ These studies stated that the measurement of salivary cortisol levels is an interesting way of testing adrenal function in infants and children, since it provides a reliable tool for the determination of the physiology and developmental characteristics of cortisol metabolism.^26^

In the present study, it was found that salivary levels of DHEAS and cortisol are greater in males than females at pre-pubertal, pubertal and post-pubertal stages (Table 6). According to similar studies^23^ ^,^ ^27^ correlating DHEA and DHEAS levels in serum to puberty, it was suggested that DHEA concentrations are higher in females than in males in almost all the age groups. Whereas average as well as median levels of DHEAS were higher in all instances in males than in females, starting from the group aged 11-15 years. Also in literature, several authors have showed the influence of BMI, body weight, age and stress on cortisol levels.^28^ ^,^ ^29^ Hence, the results found in the present study can be attributed to these factors as well. Therefore, there is a need for new studies to establish this relationship accurately. 

The major drawbacks of using it in routine clinical practice are the high cost involved with the assay procedures, which are time consuming.^16^ Also, further investigations are required to potentiate the use of salivary assay as a routine diagnostic protocol using larger sample size to establish the precise ranges of each of the six cervical vertebral stages.

## CONCLUSION

This study showed progressive increase in the salivary levels of both the biomarkers with pubertal status, with higher levels in males than females at all growth stages. Furthermore, new prospective study of skeletal maturation and salivary levels of these biomarkers will be helpful and more comprehensive.
